# Angiosarcoma Arising in an Ovarian Fibroma: A Case Report

**DOI:** 10.4061/2010/842592

**Published:** 2010-10-31

**Authors:** Eduardo Cambruzzi, Karla Lais Pegas, Daniel Marini Milani, Ricardo Pedrini Cruz, Enilde Heloena Guerra, Márcio Balbinotti Ferrari

**Affiliations:** ^1^Laboratório de Patologia, Hospital Conceição de Porto Alegre, Universidade Luterana do Brasil, B. Cristo Redentor, 91350-200 Porto Alegre, RS, Brazil; ^2^Santa Casa de Porto Alegre, Hospital de Clínicas de Porto Alegre, Brazil; ^3^Hospital Conceição de Porto Alegre, Brazil; ^4^Acadêmica de Medicina, Universidade Luterana do Brasil, Brazil

## Abstract

Primary ovarian angiosarcoma is a very rare gynaecological sarcoma, with poor prognosis. These tumors are though to arise from carcinosarcomas, teratomas, or the ovarian vasculature and occur at any age. There are only a few cases reported in the international literature, most commonly associated to surface epithelial-stromal or germ cell tumours. Herein, the authors report the clinicopathologic features of an angiosarcoma arising in an ovarian fibroma. A 65-year-old patient was admitted with a palpable mass in the hypogastrium. Grossly, the removed ovary was completely replaced by a solid tumor mass. On histological analysis, the lesion revealed the typical histological features of angiosarcoma with sinusoidal patterns and anaplastic cells, admixed with spindle-shaped cells arranged in fascicles or in a storiform pattern, compatible with a fibroma. The vascular component was strongly immunopositive for CD31 and CD34. The patient was submitted to chemotherapy, and she was alive for two months after surgical proceedings.

## 1. Introduction

Mesenchymal neoplasms of the ovary include all primary ovarian neoplasms of connective tissue origin, and not of teratomatous or surface epithelial-stromal origin. This mode of origin cannot be excluded in a number of cases in view of the possibility of one-sided differentiation of a teratoma or of a carcinosarcoma of the ovary. Mesenchymal neoplasms of the ovary can be benign or malignant and are classified on the basis of their line of differentiation. This uncommon group of neoplasms is not specific to the ovary, determining difficult problems in diagnosis, histogenesis, behavior, and therapy [1].

Angiosarcoma is a very rare malignant mesenchymal ovarian neoplasm. The tumor is usually unilateral, with uncertain histogenesis. It may originate from the vascular tissue present in the ovary or from a teratoma in which the vascular component has overgrown the other parts of the tumor. Patients usually have symptoms related to the presence of a lower abdominal mass, which may be associated with torsion of the tumor and hemorrhage [2–5]. 

Fibroma is the most common ovarian neoplasms of connective tissue origin and constitutes 3%–5% of ovarian neoplasms. The histogenesis of ovarian fibroma is controversial. The neoplasm most likely arises from mesenchymal cells of the ovarian stroma, which differentiate in the fibroblastic direction. Ovarian fibroma is bilateral in 4%–8% of patients and multiple in 10% of cases. Patients with ovarian fibroma frequently are asymptomatic, or they manifest with abdominal pain, urinary symptoms, and ascite. Fibroma of the ovary is a benign neoplasm, with excellent prognosis, and the treatment of choice is excision of the affected ovary [1, 6, 7]. 

The authors report a case of angiosarcoma arising in an ovarian fibroma, describing morphologic and immunohistochemical findings and diagnostic criteria of these two distinct neoplasms.

## 2. Clinical History

A 65-year-old female black patient was admitted with heaviness sensation in the hypogastrium for a few months. She had not any other complaint. Physical examination revealed a palpable abdominal mass in the lower abdomen. Abdominal computed tomography revealed a massive intraperitoneal lesion, extending from the pelvis to the level of L2, determining compression on small bowel and inferior vena cava and measuring 23.0 × 22.0 × 12.0 cm. With the hypothesis of ovarian neoplasm, an exploratory laparotomy was performed. A massive ascites was found (about five liters) and a brownish ovoid tumor in the topography of the right ovary, with loose adhesions in the omental and small bowel segments.

## 3. Pathological Findings

**Figure 1:**
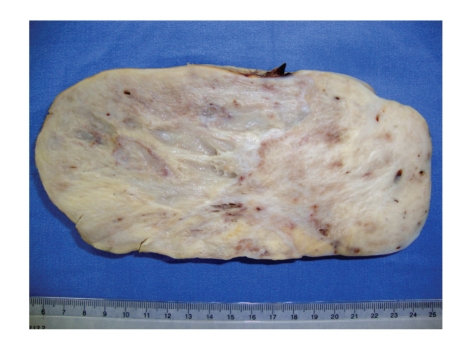
Angiosarcoma arising in an ovarian fibroma: the sectionated surface shows a yellow-white fibrous tumor with hemorrhagic zones.

**Figure 2:**
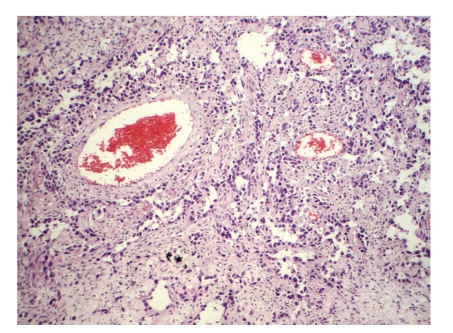
Ovarian angiosarcoma: a malignant tumor composed of vascular spaces of varying size and appearance, lined by large endothelial cells with bizarre nuclei, hematoxilin-eosin, 200x.

**Figure 3:**
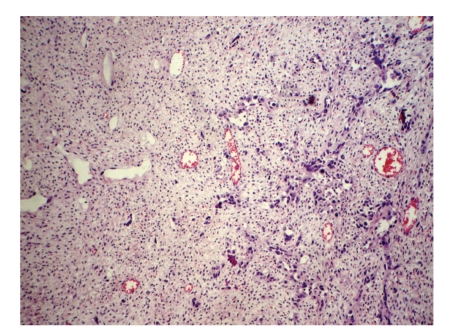
Angiosarcoma arising in an ovarian fibroma: the transition between the invasive pattern of the vascular neoplasia and the fascicular pattern of an ovarian fibroma, hematoxilin-eosin, 200x.

**Figure 4:**
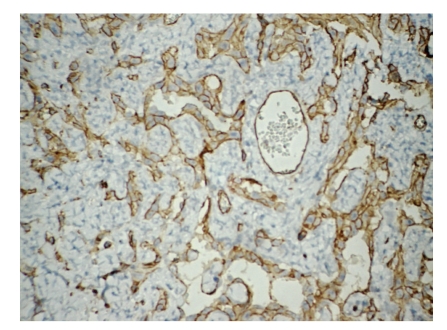
Ovarian angiosarcoma: immunohistochemical staining showed positivity for CD34, streptavidin-biotin, 200x.

The surgical specimen consisted of uterus, ovaries, tubes, greater omental, and peritoneal biopsies of the diaphragm and parietocolic gutter, previously fixed in formalin. The right ovary weighed 2825.0 g and measured 23.0 × 18.0 × 9.0 cm. On a cut section, the parenchyma was replaced by a yellowish-gray, frosted, swirled and firm tumor, with reddened areas in the middle (Figure 1). At microscopy, on hematoxilin-eosin technique, a tumor composed of two distinct cellular patterns was identified. In about 10% of the tumor, it showed hypercellular areas with mesenchymal differentiation, high mitotic index (about 10 mitotic figures per 10 high-power field) with necrotic foci, composed of epithelioid or polygonal cells with marked atypia, moderate to large size, forming vascular channels of varying size (Figure 2), invading the albuginea and even the epiploon. In the remainder of the tumor volume, a moderately cellular neoplasm was identified, with low mitotic index (about 1 mitotic figure per 10 high power fields), no evidence of necrosis, composed of spindle cells with scant cytoplasm and mild atypia, arranged in fascicles, surrounded by hyalinized or collagenized stroma (Figure 3). The two distinct cellular patterns were intimately admixed. The immunohistochemical study of lesion revealed positive expression for CD31, CD34 (Figure 4), AE1/AE3 (focal and strong), CD117 (focal and weak), Ki67 (present in about 70% of nuclei), and vimentin and negative immunostaining for desmin, smooth muscle actin (1A4), S100 protein, and CD10, in areas composed of epithelioid cells forming vascular channels. In areas composed of spindle cells, positive immunoreactivity was found only for vimentin. The left ovary had corpora albicantia and cortical epithelial inclusion cyst. 

 The morphology of ovarian lesions associated with the findings of the immunohistochemical study was consistent with angiosarcoma associated with ovarian fibroma. In the remainder of the surgical specimen, intramural, subserosal and submucosal uterine body leiomyomas, endometrial polyp, and squamous cervical intraepithelial neoplasia grade 3 (CIN 3) were identified. The surface of the albuginea was compromised by the neoplasm. Ectocervical margin was free of dysplasia. After two months of clinical followup, the patient had no evidence of disease progression, being evaluated by the oncology team and underwent chemotherapy regimen.

## 4. Discussion

Pure soft tissue sarcomas of somatic type rarely occur as primary tumors of the ovary. They tipically present as a rapidly enlarging adnexal mass. Their histological appearance is similar to soft tissue tumors in other locations. In some cases, sarcomas arise as a component of a complex ovarian tumor such as carcinosarcoma, adenosarcoma, immature teratoma, or dermoid cyst or from heterologous elements in a Sertoli-Leydig cell tumor. Rare cases of primary ovarian angiosarcoma have been reported. Ovarian sarcomas may be associated with surface epithelial-stromal tumors, particularly serous and mucinous tumors, the so-called mural nodules. These tumors must be distinguished from metastatic sarcoma to the ovary [1–5, 8]. In this paper, the authors described a case of angiosarcoma associated with an ovarian stromal/sex cord differentiation tumor, which corresponded to a fibroma, not currently being found this association in literature.

Angiosarcoma is a very rare ovarian neoplasm that recapitulates many of the functional and morphologic features of normal endothelium. Although the histogenesis of the tumor is uncertain, it may originate from the vascular tissue present in the ovary. It may vary from highly differentiated tumors that resemble hemangiomas to those in which anaplasia makes them difficult to distinguish from carcinomas. In general, angiosarcoma is a highly aggressive cutaneous tumors sometimes associated with lymphedema. Less than one quarter presents as a deep soft tissue mass [1, 9, 10]. The age of the patients with ovarian angiosarcoma varies from 19 to 77 years. The tumor usually is unilateral, but bilateral tumors have been recorded. Macroscopically, the tumors usually are large, blue-brown, hemorrhagic, soft, and friable. They may be confined to the ovary but often are associated with invasion of the surrounding structures [3, 5, 10–12].

Microscopically, angiosarcomas are composed of vascular spaces of varying size and appearance, lined by endothelial cells that are usually large, showing atypical appearance, bizarre nuclei, and mitotic activity. In some areas, the tumor may contain a considerable amount of connective tissue interspersed between the vascular spaces. Some tumors exhibit a solid pattern. The tumor invades locally and metastasizes via the bloodstream. Angiosarcoma arising in the ovary must be distinguished from immature teratomatous neoplasms with a prominent vascular component. The presence of other neoplastic germ cell elements distinguishes teratoma from primary angiosarcoma. Ovarian angiosarcoma may to be distinguished too from lymphangiosarcoma, yolk salk tumor, and leiomyosarcoma on the basis of histological appearance and immunohistochemistry. Prognosis is poor, especially in patients who have metastases at the time of presentation. The tumor has not responded to combination chemotherapy regimens. Angiosarcomas usually express the usual vascular antigens including von Willebrand factor, CD31, and CD34. Cytokeratin is expressed in about one third of soft tissue angiosarcomas. Immunopositivity to actin and laminin can be found too [2, 4, 5, 10, 13–17]. The features correlated with poor outcome include older age, invasion of adjacent structures, large size, and high Ki67 values [9]. 

Fibromas account for 4% of all ovarian tumors, and it is by far the most common sex cord-stromal tumor. They are most common in middle age and are rare in children, except for those in patients with the basal cell nevus syndrome, in whom the tumors are almost always bilateral, multinodular, and calcified. Clinically, patients with ovarian fibroma frequently are asymptomatic, mainly because of the tumor's small size. When symptoms do occur, they manifest with abdominal enlargement, urinary symptoms, and abdominal pain. Fibromas average 6 cm in diameter and are tipically uniformly solid, firm, white neoplasms. Hemorrhage, necrosis, calcification, and cyst formation may be seen. Fibromas over 10 cm in diameter are associated with ascites in up 40% of cases and Meig's syndrome (ascites and pleural effusion) in about 1% of cases [6, 7, 18].

The ovarian fibroma is composed of spindle-shaped cells with uniform, bland nuclei and scant cytoplasm that may contain small amounts of lipid or eosinophilic droplets. The cells are arranged in fascicles or in a storiform pattern. Mitoses are absent or rare. The tumor is sparsely to moderately cellular with abundant intercellular collagen, hyalinized plaques, and variable degrees of edema. In some cases, fibromas can be cellular and mitotically active. Small, irregular nests or tubules of sex cord cells are occasionally found in the stroma. The sex cord cells are polygonal and have uniform nuclei and small amounts of cytoplasm. They resemble indifferent sex cord cells or granulose cells. The absence of fat differentiates a fibroma from a thecoma, but it cannot distinguish between a fibroma and a completely fibrosed thecoma. Massive edema of the ovary and fibromatosis may resemble an edematous fibroma. Fibromas express vimentin and may be immunoreactive for alpha-inhibin, and it is adequately treated by surgical excision [6, 7, 18–20].

## 5. Conclusion

The authors emphasize the rarity of primary malignant mesenchymal neoplasms of the ovary, especially the cases of angiosarcoma, which most often are associated with teratomas or epithelial/surface tumors of the ovary. To the best of our knowledge, the reported case is peculiar morphological finding that has not been previously reported yet.
